# Isolation and Characterization of Two Postsynaptic Neurotoxins From Indian Cobra (*Naja Naja*) Venom

**DOI:** 10.3389/fphar.2022.815079

**Published:** 2022-03-28

**Authors:** Tam M. Huynh, Anjana Silva, Geoffrey K. Isbister, Wayne C. Hodgson

**Affiliations:** ^1^ Monash Venom Group, Department of Pharmacology, Biomedical Discovery Institute, Monash University, Clayton, VIC, Australia; ^2^ Department of Parasitology, Faculty of Medicine and Allied Sciences, Rajarata University of Sri Lanka, Saliyapura, Sri Lanka; ^3^ Clinical Toxicology Research Group, University of Newcastle, Newcastle, NSW, Australia

**Keywords:** *Naja naja*, snake, antivenom, neuromuscular paralysis, neurotoxin

## Abstract

The Indian Cobra (*Naja naja*) is among the “Big Four” responsible for most of the snakebite envenoming cases in India. Although recent proteomic studies suggest the presence of postsynaptic neurotoxins in *N. naja* venom, little is known about the pharmacology of these toxins. We isolated and characterized α-Elapitoxin-Nn2a (α-EPTX-Nn2a; 7020 Da) and α-Elapitoxin-Nn3a (α-EPTX-Nn3a; 7807 Da), a short-chain and long-chain postsynaptic neurotoxin, respectively, which constitute 1 and 3% of *N. naja* venom. α-EPTX-Nn2a (100–300 nM) and α-EPTX-Nn3a (100–300 nM) both induced concentration-dependent inhibition of indirect twitches and abolished contractile responses of tissues to exogenous acetylcholine and carbachol, in the chick biventer cervicis nerve-muscle preparation. The prior incubation of tissues with Indian polyvalent antivenom (1 ml/0.6 mg) prevented the *in vitro* neurotoxic effects of α-EPTX-Nn2a (100 nM) and α-EPTX-Nn3a (100 nM). The addition of Indian polyvalent antivenom (1 ml/0.6 mg), at the t_90_ time point, could not reverse the *in vitro* neurotoxicity of α-EPTX-Nn2a (100 nM). The *in vitro* neurotoxicity of α-EPTX-Nn3a (100 nM) was partially reversed by the addition of Indian polyvalent antivenom (1 ml/0.6 mg), as well as repeated washing of the tissue. α-EPTX-Nn2a displayed non-competitive antagonism of concentration-response curves to carbachol, with a pA_2_ of 8.01. In contrast, α-EPTX-Nn3a showed reversible antagonism of concentration-response curves to carbachol, with a pA_2_ of 8.17. *De novo* sequencing of α-EPTX-Nn2a and α-EPTX-Nn3a showed a short-chain and long-chain postsynaptic neurotoxin, respectively, with 62 and 71 amino acids. The important observation made in this study is that antivenom can reverse the neurotoxicity of the clinically important long-chain neurotoxin, but not the short-chain neurotoxin, from *N. naja* venom.

## 1 Introduction

The Indian cobra (*Naja naja*) is among the “Big Four” responsible for most human snake envenoming cases in India (Simpson & Norris, 2007). It is distributed throughout Sri Lanka, India, Pakistan, Southern Nepal and Bangladesh. Envenoming by *N. naja* causes life-threatening neurotoxicity and severe local necrosis, and is treated by administration of Indian polyvalent antivenom (Kularatne et al., 2009; Mohapatra et al., 2011).

Although proteomic and transcriptomic investigations provide insights into the toxin composition of *N. naja* venom (Tasoulis & Isbister, 2017), little is known about the pharmacology of most of these toxins. Neuromuscular paralysis is a well-known sequalae of envenoming by *N. naja*, so the pharmacological characterization of the neurotoxins with potential clinical relevance is of high importance. Recent proteomic studies suggest the dominance of three-finger toxins (3FTx) in the composition of *N. naja* venom from India and Sri Lanka (Senji Laxme et al., 2021; Wong et al., 2021). These toxins include both the neurotoxic and cytotoxic 3FTx’s, which have varying relative abundances depending on the biogeographic region of the venom. The percentage of neurotoxic 3FTx’s in *N. naja* venom from India can range between 30 and 80% (Senji Laxme et al., 2021). Despite these disparities, postsynaptic neurotoxins are the only toxins in *N. naja* venom that cause neuromuscular paralysis (Wong et al., 2021), and neurotoxicity seen in envenomed patients could be entirely attributed to the postsynaptic neurotoxins. Postsynaptic neurotoxins inhibit neurotransmission at the neuromuscular junction by competitively binding with the two acetylcholine binding sites of the nicotinic acetylcholine receptors (nAChRs) of the motor-end plate (Barber et al., 2013). Postsynaptic neurotoxins are further classified as short-chain or long-chain neurotoxins based on their structural and functional differences (Barber et al., 2013; Silva et al., 2018). Although both types of postsynaptic neurotoxins can functionally antagonize the human nAChR, the higher potency and lower reversibility of long-chain neurotoxins make them more clinically important in human neuromuscular paralysis (Silva et al., 2018).

Several postsynaptic neurotoxins have been isolated from the venom of *N. naja*, for various parts of its geographical distribution, to investigate their mode of action (Meldrum, 1965; Charles et al., 1981; Charles et al., 1982; Wong et al., 2016) and neutralization by antivenom (Wong et al., 2016). However, their antagonistic potency and biochemical properties have not been determined. This study aimed to isolate and pharmacologically characterize the main neurotoxins from *N. naja* venom from Sri Lanka, and to determine the *in vitro* efficacy of Indian Polyvalent antivenom against these toxins.

## 2 Materials and Methods

### 2.1 Venoms and Antivenoms

Pooled, freeze-dried *N. naja* venom from Sri Lanka was used for this study. Indian polyvalent antivenom was purchased from VINS Byproducts (Andra Pradesh, India; Batch No: 01AS11005, expiry date: 13/02/2023). According to the manufacturer’s instructions, 1 ml of the antivenom neutralizes 0.6 mg of *N. naja* venom. The amount of antivenom required for neutralizing the *in vitro* neurotoxicity of the isolated toxin was calculated based on the relative abundance of the toxin in the whole venom.

### 2.2 Chemical and Reagents

The following chemicals and drugs were used: acetylcholine chloride (Sigma-Aldrich, St. Louis, MO, United States), acetonitrile (ACN, Merck, Darmstadt, Germany), ammonium bicarbonate (Sigma-Aldrich, St. Louis, MO, United States), carbamylcholine chloride (carbachol; Sigma-Aldrich, St. Louis, MO, United States), dithiothretiol (Merck, Darmstadt, Germany), formic acid (Sigma-Aldrich, St. Louis, MO, United States), iodoacetamide (GE Healthcare, Uppsala, Sweden), LCMS grade acetonitrile (Fisher Scientific, Loughborough, United Kingdom), potassium chloride (KCl, Ajax Finechem Pty. Ltd., Taren Point, Australia), proteomics grade bovine trypsin (Sigma-Aldrich, St. Louis, MO, United States), trifluoroacetic acid (TFA, Auspep, Melbourne, Australia), d-tubocurarine (Sigma-Aldrich, St. Louis, MO, United States) and trifluroethanol (Sigma-Aldrich, St. Louis, MO, United States). Unless otherwise indicated, all chemicals were dissolved or diluted in milli-Q water.

### 2.3 Isolation and Purification of Toxins

Chromatography was performed using a high-performance liquid chromatography (HPLC) system (Shimadzu, Kyoto, Japan). Freeze-dried *N. naja* venom (2 mg) was reconstituted in 500 μl milli-Q water (Millipore Corporation, Billerica, MA, United States) and centrifuged at 12,000 rpm for 10 min before being loaded into a Phenomenex Jupiter semi-preparative C18 column (5 μm, 250 mm × 10 mm; Phenomenex, Torrance, CA, United States) that was equilibrated with solvent A (0.1% TFA) at a flow rate of 2 ml/min. The fractions were then eluted using the following gradient of solvent B (90% ACN in 0.09% TFA); 0–25% over 10 min, 25%–80% between 10 and 60 min and 80–0% between 60 and 65 min. The eluent was monitored at 214 nm. Fractions were collected manually according to the peaks in the chromatogram and freeze-dried immediately, then later screened using the chick biventer cervicis nerve-muscle preparation to identify fractions displaying neurotoxicity.

### 2.4 Mass Spectrometry and Amino Acid Sequencing

#### 2.4.1 Intact Protein Analysis With Matrix Associated Laser Desorption Time of Flight (MALDI-TOF) Mass Spectrometry

Protein samples were analysed by LC-MS using a quadrupole TOF mass spectrometer (MicroTOFq, Bruker Daltonics, Bremen, Germany) coupled online with a 1,200 series capillary HPLC (Agilent technologies, Santa Clara, CA, United States). Samples were injected onto a MabPac SEC-1 5 um 300 A 50 × 4 mm (Thermo Scientific) column with 50% acetonitrile 0.05% TFA, 0.05% FA at a flow rate of 50 μl/min. The protein was eluted over a 20 min run-time monitored by UV detection at 254 nm. The eluant was nebulised and ionised using the Bruker electrospray source with a capillary voltage of 4500 V dry gas at 180°C, flow rate of 4 L/min and nebuliser gas pressure at 300 mbar. After 20 min, the flow path was switched to infuse Low concentration Tune mix (Agilent technologies, Santa Clara, CA, United States) to calibrate the spectrum post acquisition. The spectra were extracted and deconvoluted using Data explorer software version 3.4 build 192 (Bruker Daltonics, Bremen, Germany).

#### 2.4.2 Electrospray-Ionisation Coupled With Mass-Spectrometry/Mass Spectrometry (ESI-LCMS/MS)

The sample (2.5–5 μg) was buffer exchanged into 50 mM ammonium bicarbonate and reduced in 2.5 mM DTT at 60°C for 5 min followed by alkylation with 10 mM chloroacetamide for 30 min at room temperature. The enzyme was then added at the rate of 0.5 μg per 10 μg of protein and incubated at 37°C overnight. For chymotrypsin digests 50 mM Tris buffer was used in place of ammonium bicarbonate.

All enzyme digests were analysed by LC-MS/MS using the QExactive mass spectrometer (Thermo Scientific, Bremen, Germany) coupled online with a RSLC nano HPLC (Ultimate 3,000, Thermo Scientific, Bremen, Germany). Sample (200 ng) was injected and concentrated on a 100 μm, 2 cm nanoviper pepmap100 trap column with 97.5% buffer A (0.1% TFA) at a flow rate of 15 μl/min. The peptides then eluted and separated with a Thermo RSLC pepmap100, 75 μm × 50 cm, 100Ǻ pore size, reversed phase nano column with a 30 min gradient of 92.5% buffer A (0.1% formic acid) to 42.5% B (80% ACN 0.1% formic acid), at a flow rate of 250 nl/min. The eluant was nebulised and ionised using the Thermo nano electrospray source with a distal coated fused silica emitter (New Objective, Woburn, MA, United States) with a capillary voltage of 1900 V. Peptides were selected for MSMS analysis in Full MS/dd-MS2 (TopN) mode with the following parameter settings: TopN 10, resolution 70,000, MSMS AGC target 5e5, 118 ms Max IT, NCE 27, 1.8 m/z isolation window, dynamic exclusion was set to 10 s.

#### 2.4.3 De novo Protein Sequencing

Data from the LCMS/MS acquisitions was analysed using Peaks AB Ver. 2.0 (Bioinformatics Solutions Inc., Ont, Canada), to derive a *de novo* protein sequence. The following search parameters were used: missed cleavages, 2; peptide mass tolerance, ± 10 ppm; peptide fragment tolerance, ± 0.05 Da; fixed modifications, carbamidomethyl (Cys); Variable modification, oxidation (Met).

Sequences were validated using Byonic (ProteinMetrics) V 3.1–19 and a precursor and fragment mass tolerance of 20 ppm. Modifications specified were Carbamidomethyl @C fixed and Oxidation @M Variable common 1. The protein output was set to 1% FDR.

### 2.5 Chick Biventer Cervicis Nerve-Muscle Preparation

Male chickens (aged 4–10 days) were killed by exsanguination following CO_2_ inhalation. Biventer cervicis nerve-muscle preparations were dissected and then mounted on wire tissue holders under 1 g resting tension in 5 ml organ baths. Tissues were maintained at 34°C, bubbled with 95% O_2_ and 5% CO_2_, in physiological salt solution of the following composition (mM): 118.4 NaCl, 4.7 KCl, 1.2 MgSO_4_, 1.2 KH_2_PO_4_, 2.5 CaCl_2_, 25 NaHCO_3_ and 11.1 glucose. Indirect twitches were evoked by stimulating the motor nerve (0.1 Hz; 0.2 ms) at supramaximal voltage (10–20 V), using a Grass S88 stimulator (Grass Instruments, Quincy, MA). Selective stimulation of the nerve was confirmed by the abolishment of twitches by the addition of d-tubocurarine (10 μM). Tissues were then repeatedly washed with physiological salt solution to restore twitch response to nerve stimulation. Contractile responses of the tissues to exogenous acetylcholine (ACh; 1 mM for 30 s), carbachol (CCh; 20 μM for 60 s) and KCl (40 mM for 30 s) were obtained in the absence of nerve stimulation. Nerve stimulation was then recommenced for at least 30 min before the addition of the toxin or antivenom.

To examine the ability of antivenom to neutralise the toxins (i.e., prevention study), tissues were equilibrated with antivenom for 10 min before the toxin was added. To determine the ability of antivenom to reverse the toxin induced neurotoxicity (i.e., reversal study), antivenom was added at t_90_ (i.e., time at which the initial twitch height was inhibited by 90%). If the antivenom partially restored the toxin induced neurotoxicity, the reversible nature of the binding of the toxin to skeletal nAChR was assessed in subsequent experiments by repeatedly washing the organ bath for a period of 10 s every 5 min until any recovery of twitch responses had plateaued.

At the conclusion of each experiment, ACh, CCh and KCl were re-added as above. Twitch responses and responses to exogenous agonists were measured via a Grass FT03 force displacement transducer and recorded on a PowerLab system (ADInstruments Pty Ltd., Australia).

### 2.6 Cumulative Concentration-Response Curves to Carbachol

In order to determine the potency (i.e., pA_2_ value) of the toxins, cumulative concentration–response curves to CCh (0.6–80 µM) were obtained by adding increasing concentrations of the agonist to unstimulated chick preparations without washing the preparation between each addition. After the maximum response was achieved, the tissue was repeatedly washed and allowed to recover for 30 min. Then either toxin (1–30 nM) or vehicle (milli-Q water) was allowed to equilibrate with the tissue for 60 min before the cumulative concentration–response curve to CCh was repeated in the presence of toxin or vehicle.

### 2.7 Data Analysis

The quantity of each toxin in the whole venom was determined by measuring the area under the curve of the reverse-phase HPLC and ion-exchange chromatograms with the single peak representing either toxin being expressed as a percentage of the total area under the curve of whole venom.

Nerve-mediated twitch responses and responses to ACh (30 s), CCh (60 s) and KCl (30 s) were measured *via* a Grass FT03 force displacement transducer and recorded on a PowerLab system (ADInstruments Pty Ltd., Australia). Post-venom/toxin responses were expressed as a percentage of their initial responses. An unpaired *t*-test or one-way analysis of variance (ANOVA) was used to compare the effect on twitch height of different pre-treatments. Comparison of responses to exogenous agonists before and after pre-treatment was made using a Student’s paired *t*-test. In order to determine the antagonist potency (i.e., pA_2_) of the toxin the shifts in the cumulative concentration-response curves to CCh, in the absence or presence of the toxin, were analysed using the modified Lew Angus method. All ANOVAs were followed by a Bonferroni’s multiple comparison post-hoc test. Data presented are in the form of mean ± standard error of the mean (SEM) of n experiments. All data and statistical analyses were performed using PRISM 9.2.0 (GraphPad Software, San Diego, CA, United States, 2016). For all statistical tests, *p* < 0.05 was considered statistically significant.

## 3 Results

### 3.1 *In Vitro* Neurotoxicity of Whole Venom

**FIGURE 1:**
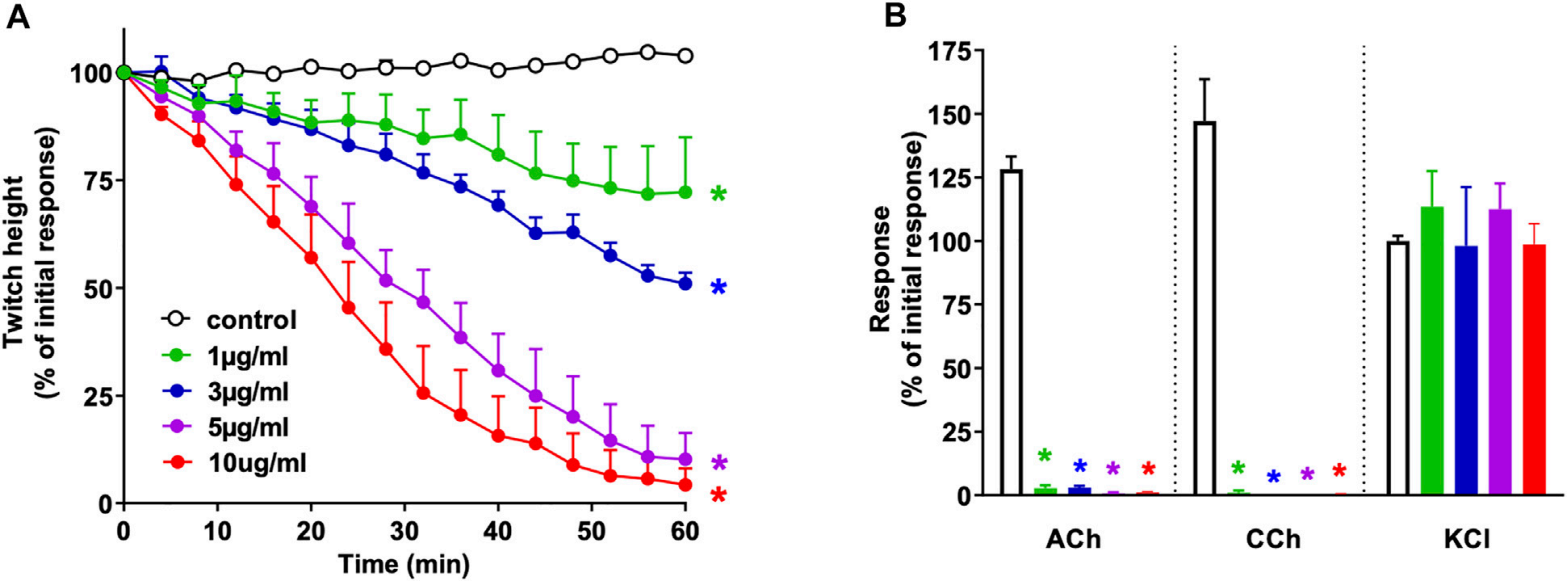
Concentration-dependent *in vitro* neurotoxicity of *N. naja* venom (1–10 μg/ml) on **(A)** indirect twitches and **(B)** responses to exogenous agonists ACh (1 mM), CCh (20 µM) and KCl (40 mM) in the chick biventer cervicis nerve-muscle preparation. *p < 0.05, significantly different from **(A)** control at 60 min (one-way ANOVA; *n* = 4) or **(B)** pre-toxin response to same agonist (paired *t*-test, *n* = 4).

*N. naja* venom (1–10 μg/ml) induced concentration-dependent inhibition of indirect twitches in the chick biventer cervicis nerve-muscle preparation (*n* = 4; *p* < 0.05, one-way ANOVA; Figure 1A). All concentrations of the venom also reduced contractile responses of tissues to exogenous ACh and CCh (*n* = 4; *p* < 0.05, paired *t*-test; Figure 1B), but not KCl, indicative of postsynaptic action at the neuromuscular junction. The t_90_, i.e., time to inhibit indirect twitches by 90%, for the venom at 10 μg/ml was 42 ± 7 min.

### 3.2 Fractionation of Venom *via* Reverse-phase HPLC and Ion-Exchange Chromatography

**FIGURE 2:**
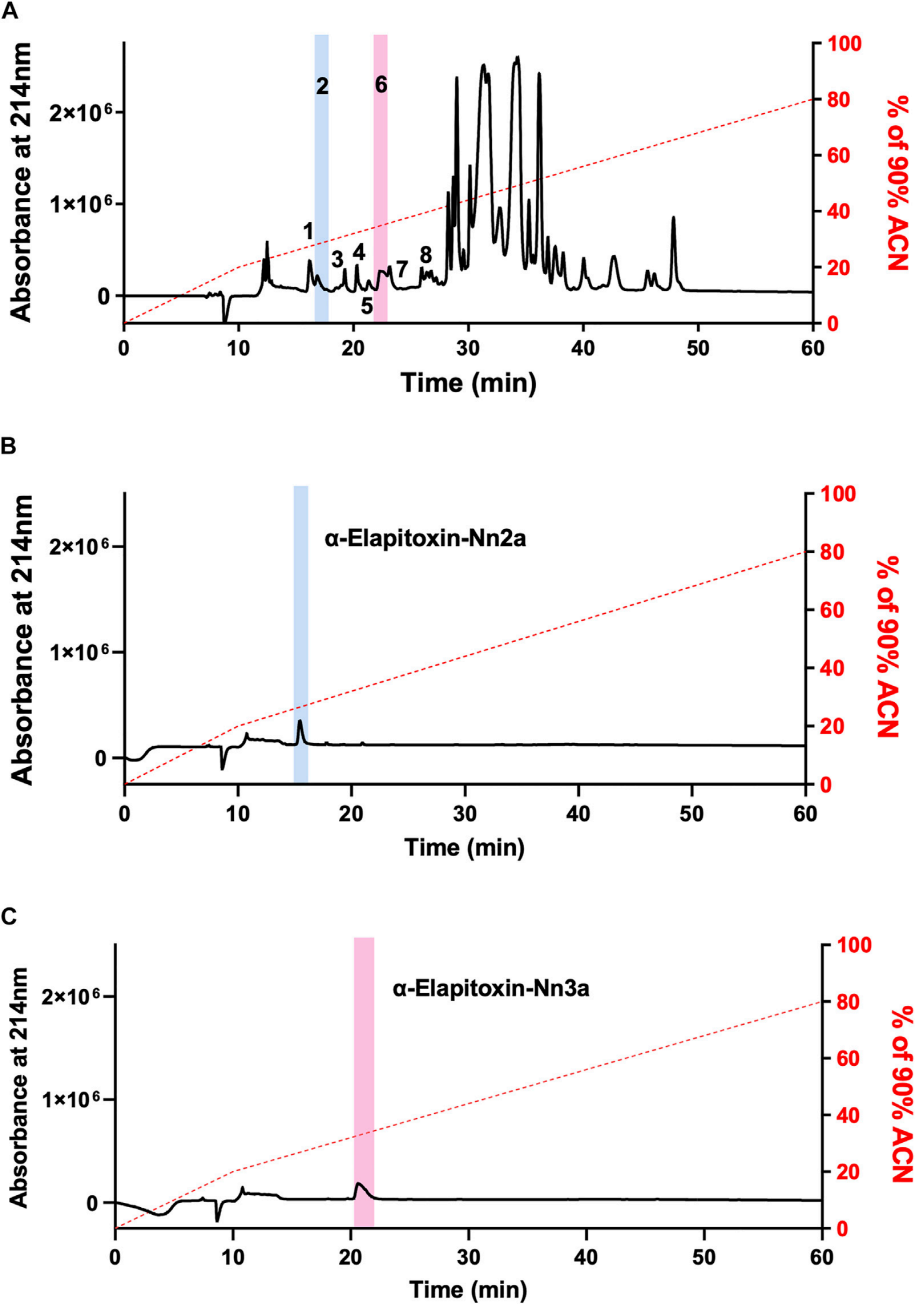
Chromatograms of *N. naja* venom showing the fractionation steps to purify α-Elapitoxin-Nn2a and α-Elapitoxin-Nn3a. **(A)** *N. naja* venom, **(B)** α-Elapitoxin-Nn2a and **(C)** α-Elapitoxin-Nn3a by reverse-phase HPLC on a Jupiter C18 semi-preparative column.

Fractionation of *N. naja* venom by reverse-phase HPLC on a Jupiter C18 semi-preparative column produced several peaks (Figure 2A). Initial screening of all fractions in the chick biventer nerve-muscle preparation showed that peak 1, peak 2 and peak 6, eluting at 15, 16 and 21 min respectively, displayed postsynaptic neurotoxicity. Peak 2 and Peak 6, subsequently named α-Elapitoxin-Nn2a and α-Elapitoxin-Nn3a respectively, were subjected to further purification using reverse-phase chromatography, both resulting in a clean peak (Figure 2B and Figure 2C). α-Elapitoxin-Nn2a and α-Elapitoxin-Nn3a are estimated to constitute approximately 1 and 3% of the whole venom protein content, respectively. The neurotoxins within peak 1 (see Supplementary Data S1) could not be further purified using the current chromatographic methods.

### 3.3 Intact Protein Analysis With MALDI-TOF Mass Spectrometry

**FIGURE 3:**
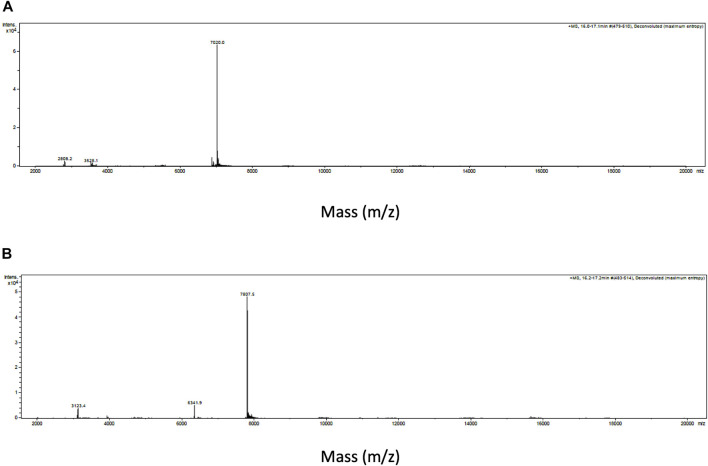
Intact protein MALDI-TOF chromatogram of **(A)** α-Elapitoxin-Nn2a indicating an intact mass of 7,020.0 Da and **(B)** α-Elapitoxin-Nn3a indicating an intact mass of 7,807.5 Da.

Intact protein analysis of α-Elapitoxin-Nn2a and α-Elapitoxin-Nn3a using MALDI-TOF both showed a single mass with molecular weights of 7,020.0 Da and 7,807.5 Da, respectively (Figure 3).

### 3.4 De novo Protein Sequencing and Protein Identification

Enzyme digest using ESI-LCMS/MS generated the following full amino acid sequence of α-Elapitoxin-Nn2a (62 amino acids) which was validated using Byonic (ProteinMetrics) V 3.1–19.

LECHNQQSSQTPTTTDCSGGETNCYKKWWSDHRGTIIERGCGCPTVKKGIELNCCTTDRCNN.

**FIGURE 4:**
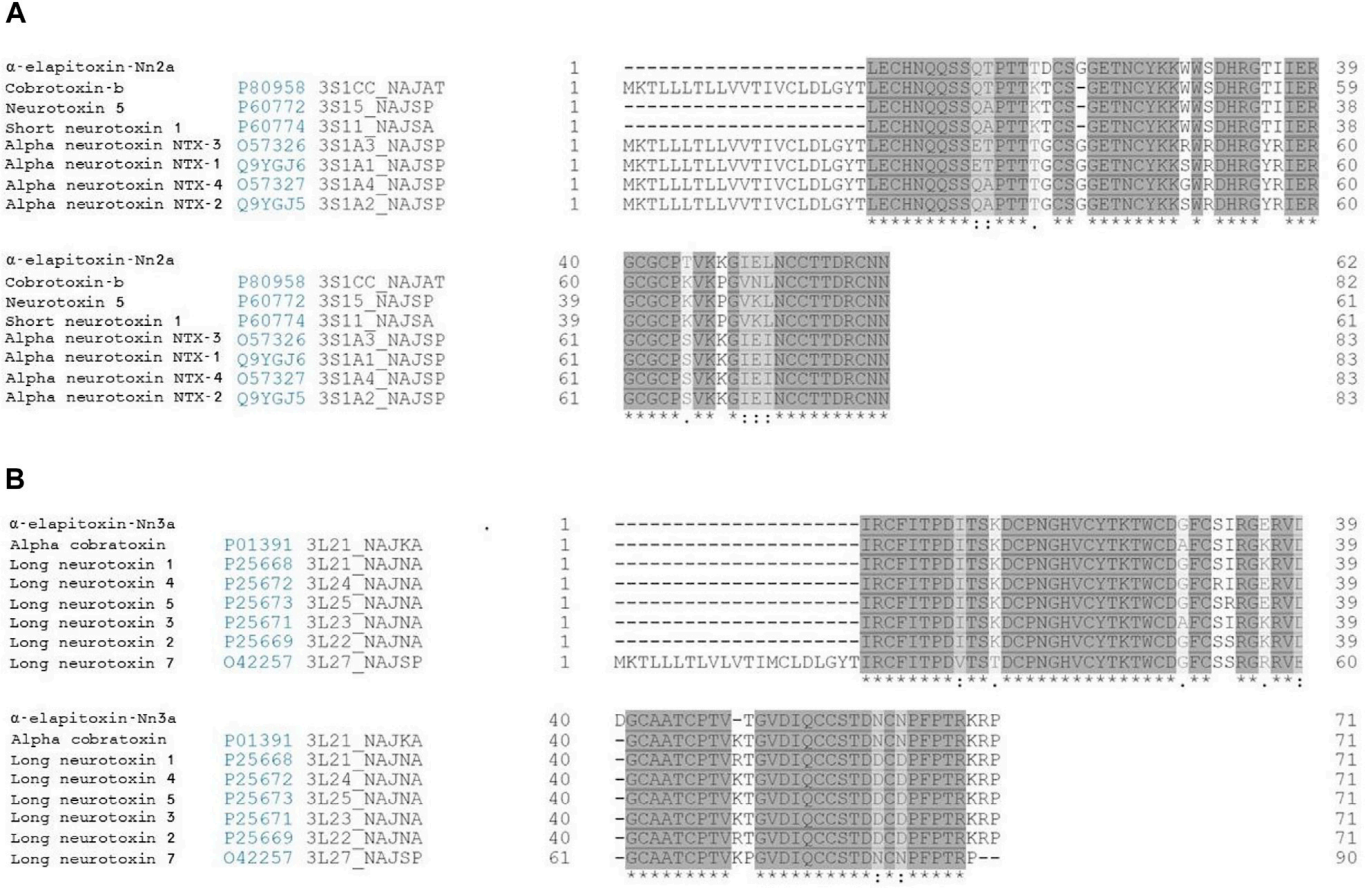
Sequence alignment (from BLAST search) of **(A)** α-Elapitoxin-Nn2a with short-chain α-neurotoxins and **(B)** α-Elapitoxin-Nn3a with long-chain α-neurotoxins from *N. naja* venom. Amino acids with (*) are fully conserved in all toxins, conserved amino acids with (.) are weakly similar properties group and amino acids with (:) are strongly similar properties group.

Fraction 2 was assigned the name “α-Elapitoxin-Nn2a” based on the rational nomenclature for naming peptide toxins from venomous animals (King et al., 2008). α-Elapitoxin-Nn2a had more than 87% sequence identity with the following short-chain α-neurotoxins, Cobrotoxin-b (*N. atra*), Neurotoxin 5 (*N. sputatrix*), Short neurotoxin 1 (*N. samarensis*), α-neurotoxin NTX-3 (*N. sputatrix*), α-neurotoxin NTX-1 (*N. sputatrix*), α-neurotoxin NTX-4 (*N. sputatrix*), α-neurotoxin NTX-2 (*N. sputatrix*) (Figure 4A).

Enzyme digest using ESI-LCMS/MS generated the following full amino acid sequence of α-Elapitoxin-Nn3a (71 amino acids) which was validated using Byonic (ProteinMetrics) V 3.1–19.

IRCFITPDITSKDCPNGHVCYTKTWCDGFCSIRGERVDLDGCAATCPTVTGVDIQCCSTDNCNPFPTRKRP.

Fraction 6 was assigned the name “α-Elapitoxin-Nn3a” based on the rational nomenclature for naming peptide toxins from venomous animals (King et al., 2008). α-Elapitoxin-Nn3a had more than 87% sequence identity with the following long chain α-neurotoxins, α-cobratoxin (*N. kaouthia*), Long neurotoxin 1 (*N. naja*), Long neurotoxin 4 (*N. naja*), Long neurotoxin 5 (*N. naja*), Long neurotoxin 3 (*N. naja*), Long neurotoxin 2 (*N. naja*) and Long neurotoxin 7 (*N. naja*) (Figure 4B).

### 3.5 *In Vitro* Neurotoxicity of α-Elapitoxin-Nn2a and α-Elapitoxin-Nn3a

#### 3.5.1 Concentration-dependent Inhibition of Twitches and Exogenous Agonists Responses

**FIGURE 5:**
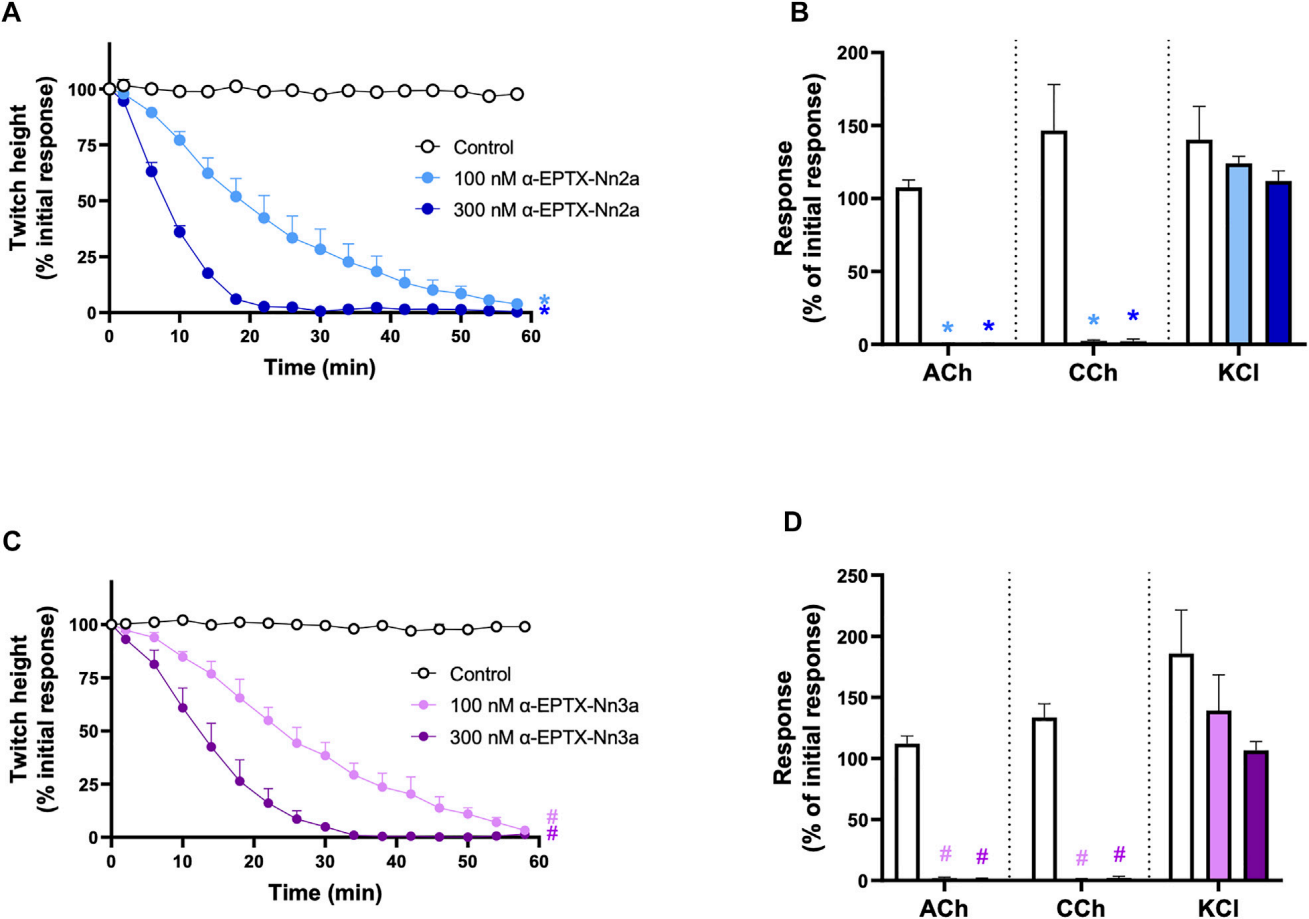
The concentration-dependent *in vitro* neurotoxic effects of; α-Elapitoxin-Nn2a (100–300 nm) on **(A)** indirect twitches and **(B)** responses to exogenous agonists ACh (1 mM), CCh (20 µM) and KCl (40 mM); and α-Elapitoxin-Nn3a on **(C)** indirect twitches and **(D)** responses to exogenous agonists ACh (1 mM), CCh (20 µM) and KCl (40 mM) in the chick biventer cervicis nerve-muscle preparation. *p < 0.05, significantly different from **(A)** control at 60 min (one-way ANOVA; *n* = 5) or **(B)** response to same agonist before α-Elapitoxin-Nn2a addition (paired *t*-test, *n* = 5). #*p* < 0.05, significantly different from **(C)** control at 60 min (one-way ANOVA; *n* = 5) or **(D)** response to same agonist before α-Elapitoxin-Nn3a addition (paired *t*-test, *n* = 5).

α-Elapitoxin-Nn2a (100–300 nM) and α-Elapitoxin-Nn3a (100–300 nM) both induced concentration-dependent inhibition of indirect twitches in the chick biventer cervicis nerve-muscle preparation (*n* = 5; *p* < 0.05, one-way ANOVA; Figure 5A and Figure 5C). Both concentrations of α-Elapitoxin-Nn2a and α-Elapitoxin-Nn3a inhibited responses of tissues to exogenous ACh and CCh (*n* = 5; *p* < 0.05, paired *t*-test; Figures 5B,D), but not KCl, indicative of a postsynaptic action at the neuromuscular junction. The t_90_ values for α-Elapitoxin-Nn2a (300 nM) and α-Elapitoxin-Nn3a (300 nM) were 16 ± 1 min and 23 ± 4 min, respectively.

#### 3.5.2 *In Vitro* Neurotoxicity Antivenom Prevention Study

**FIGURE 6:**
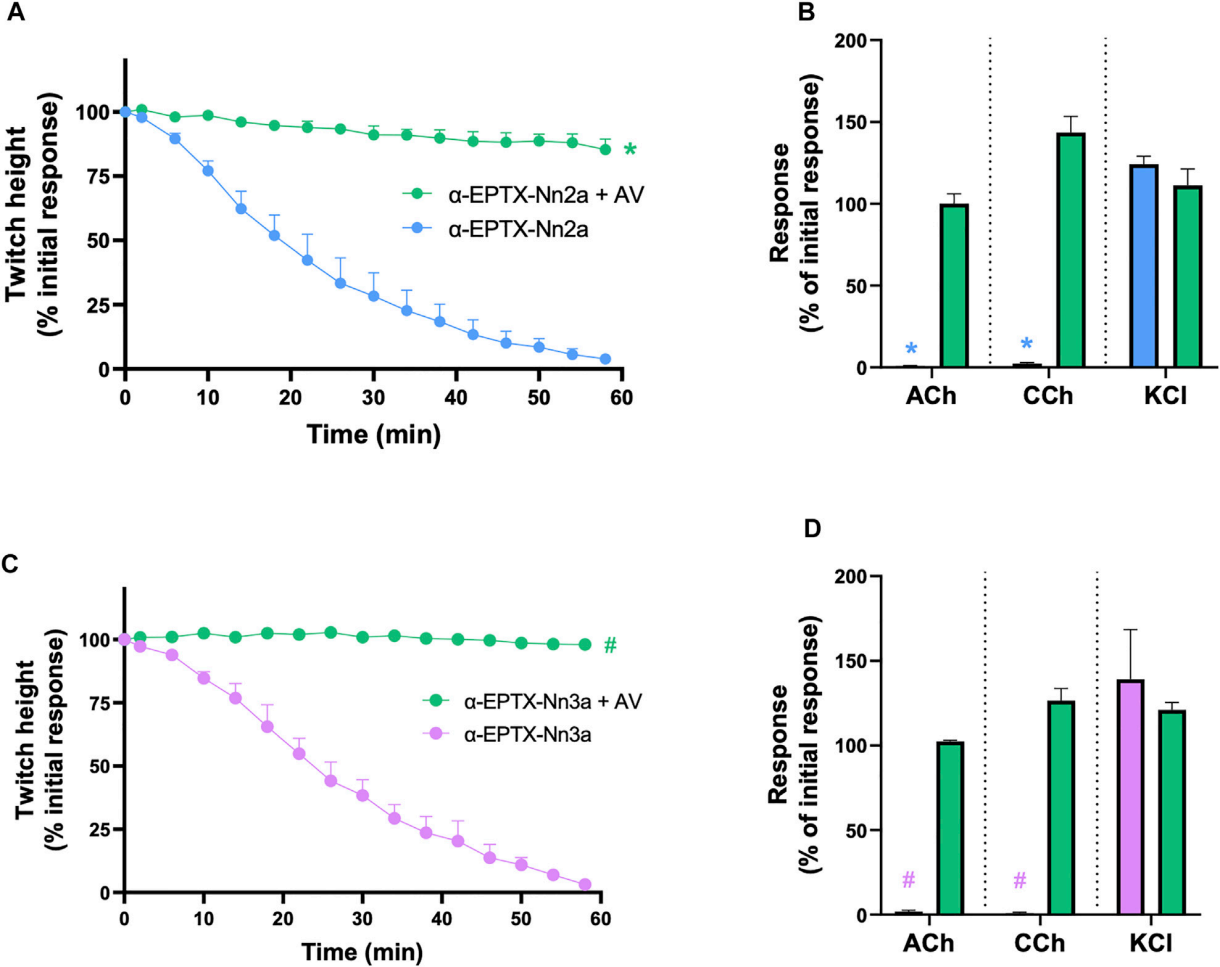
The effect of pre-addition of Indian Polyvalent antivenom (AV; at the recommended dose; 1 ml/0.6 mg) on the neurotoxicity of α-Elapitoxin-Nn2a (100 nM) and α-Elapitoxin-Nn3a (100 nM). Panels **(A)** and **(C)** show that the antivenom prevents the neurotoxicity of both α-Elapitoxin-Nn2a and α-Elapitoxin-Nn3a, respectively. Panels **(B)** and **(D)** show that the antivenom prevents the reduction of responses to ACh (1 mM) and CCh (20 µM) in the chick biventer cervicis nerve-muscle preparation by α-Elapitoxin-Nn2a and α-Elapitoxin-Nn3a, respectively. The response to KCl (40 mM) is unaffected by α-Elapitoxin-Nn2a and α-Elapitoxin-Nn3a. *p < 0.05, significantly different from **(A)** α-Elapitoxin-Nn2a alone at 60 min (unpaired *t*-test; *n* = 5) or **(B)** response to same agonist before α-Elapitoxin-Nn2a addition (paired *t*-test, *n* = 5). #*p* < 0.05, significantly different from **(C)** α-Elapitoxin-Nn3a alone at 60 min (unpaired *t*-test; *n* = 5) or **(D)** response to same agonist before α-Elapitoxin-Nn3a addition (paired *t*-test, *n* = 5).

The prior incubation of tissues with Indian polyvalent antivenom (1 ml/0.6 mg) prevented the reduction of indirect twitches by α-Elapitoxin-Nn2a and α-Elapitoxin-Nn3a (100 nM; n = 5; *p* < 0.05, unpaired *t*-test, Figure 6A and Figure 6C) and prevented the inhibition of contractile responses to ACh and CCh (*n* = 5; *p* < 0.05, unpaired, Figure 6B and Figure 6D).

#### 3.5.3 *In Vitro* Neurotoxicity Antivenom Reversal and Washing Study

**FIGURE 7:**
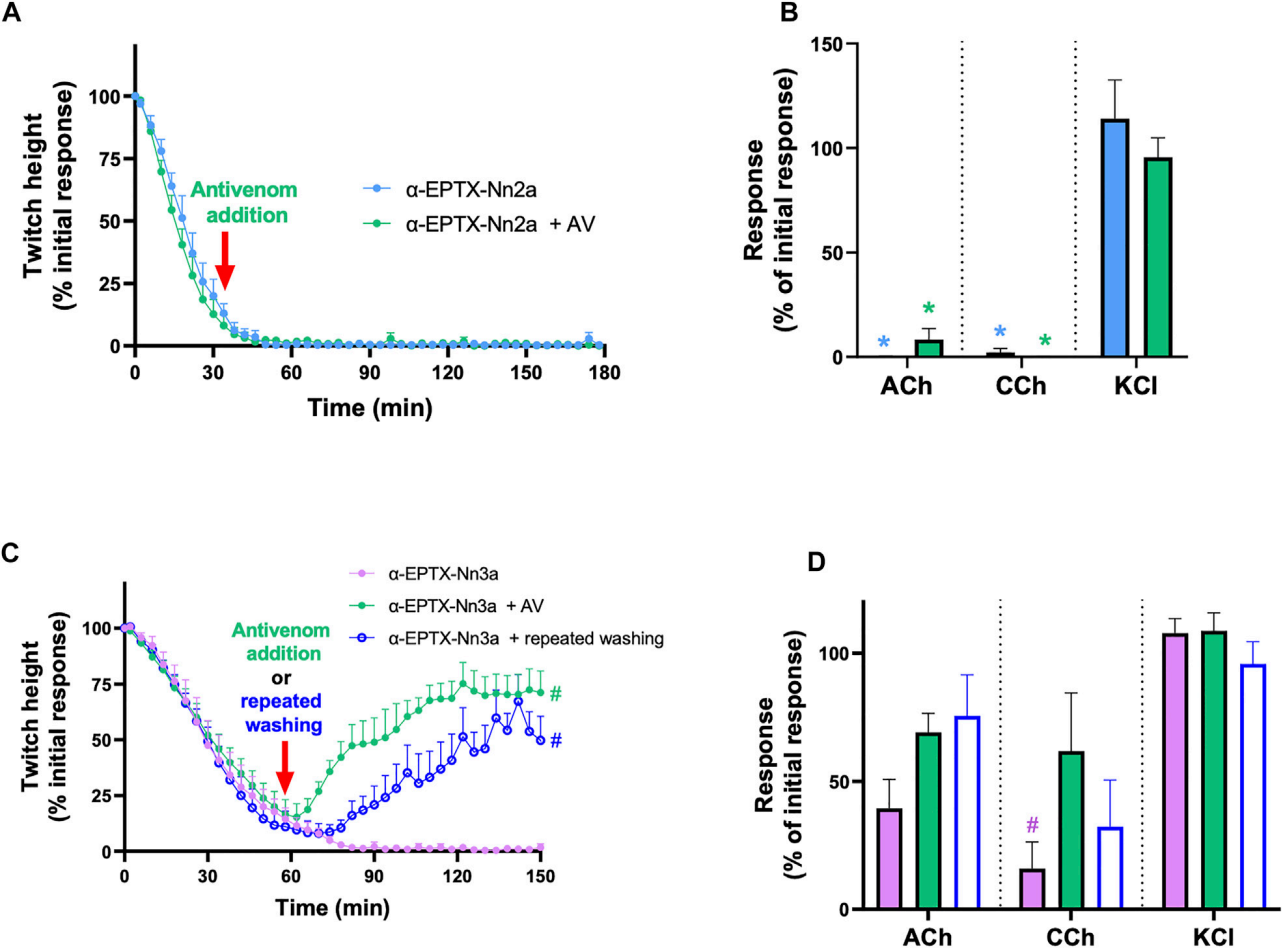
The effect of addition of Indian Polyvalent antivenom (AV; at the recommended dose; 1 ml/0.6 mg) or repeatedly washing the tissue for 10 s every 5 min at the t_90_ time point on the neurotoxicity of α-Elapitoxin-Nn2a (100 nM) and α-Elapitoxin-Nn3a (100 nM). The addition of antivenom did not affect the inhibition of **(A)** indirect twitches and **(B)** responses to exogenous agonists ACh (1 mM) and CCh (20 µM) in the chick biventer cervicis nerve-muscle preparation by α-Elapitoxin-Nn2a. The inhibition of **(A)** indirect twitches and **(B)** responses to exogenous agonists ACh (1 mM) and CCh (20 µM) the chick biventer cervicis nerve-muscle preparation by α-Elapitoxin-Nn3a, were partially reversed by both addition of antivenom or repeatedly washing the tissue. The response to KCl (40 mM) is unaffected by α-Elapitoxin-Nn2a and α-Elapitoxin-Nn3a. *p < 0.05, significantly different from **(A)** α-Elapitoxin-Nn2a alone at 180 min (unpaired *t*-test; *n* = 5) or **(B)** response to same agonist before α-Elapitoxin-Nn2a addition (paired *t*-test, *n* = 5). #*p* < 0.05, significantly different from **(C)** α-Elapitoxin-Nn3a alone at 150 min (unpaired *t*-test; *n* = 5) or **(d)** response to same agonist before α-Elapitoxin-Nn3a addition (paired *t*-test, *n* = 5).

The inhibition of indirect twitches (*n* = 5; Figure 7A) and contractile responses to ACh and CCh (*n* = 5; *p* < 0.05, paired *t*-test; Figure 7B), by α-Elapitoxin-Nn2a (100 nM), could not be reversed by the addition of Indian polyvalent antivenom (1 ml/0.6 mg), at the t_90_ time point following the addition of α-Elapitoxin-Nn2a.

The addition of Indian polyvalent antivenom (1 ml/0.6 mg), at the t_90_ time point following the addition of α-Elapitoxin-Nn3a (100 nM), partially restored the twitch responses to 71 ± 10% (*n* = 5; *p* < 0.05, one-way ANOVA; Figure 7C) of the pre-toxin twitch height by the 150 min time point. The toxin-induced reduction of responses to ACh and CCh was also significantly prevented by the delayed addition of antivenom (*n* = 5; *p* < 0.05, paired *t*-test; Figure 7D).

Repeatedly washing the tissue for 10 s every 5 min, commencing at the t_90_ time point following addition of α-Elapitoxin-Nn3a (100 nM), allowed partial recovery of the twitch responses to 50 ± 11% (*n* = 5; *p* < 0.05, one-way ANOVA; Figure 7C). Repeated washing also significantly reversed the inhibition of responses to ACh and CCh (*n* = 5; *p* < 0.05, paired *t*-test; Figure 7D).

### 3.6 Carbachol Cumulative Concentration-Response Curves

**FIGURE 8:**
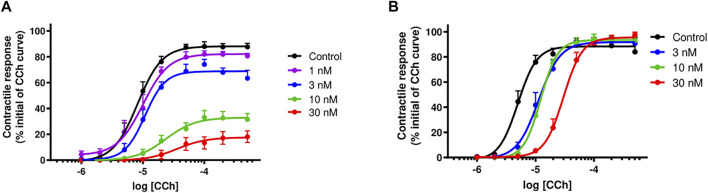
Concentration-dependent effects of **(A)** α-Elapitoxin-Nn2a (1–30 nM; *n* = 5) and **(B)** α-Elapitoxin-Nn3a (3–30 nM; *n* = 5) on cumulative concentration-response curves to carbachol (CCh) in the unstimulated chick biventer cervicis nerve-muscle preparation.

α-Elapitoxin-Nn2a (1–30 nM; *n* = 5) caused a concentration-dependent, non-parallel shift of the cumulative concentration-response curve to CCh, with a concentration-dependent reduction of the maximum response in unstimulated chick biventer cervicis nerve-muscle preparations (Figure 8A). This indicates that α-Elapitoxin-Nn2a is acting as a non-competitive antagonist at the skeletal nAChR. Using the modified Lew and Angus method, the pA_2_ value of α-Elapitoxin-Nn2a was calculated to be 8.01 (95% CI: 7.74–8.23).

α-Elapitoxin-Nn3a (3–30 nM; *n* = 5) caused a concentration-dependent parallel shift of the cumulative concentration-response curve to CCh, without any reduction of the maximum response in unstimulated chick biventer cervicis nerve-muscle preparations (Figure 8B). This shows that the antagonism of α-Elapitoxin-Nn3a at the skeletal nAChR is reversible (i.e., competitive) in nature. Using the modified Lew and Angus method, the pA_2_ value of α-Elapitoxin-Nn3a was calculated to be 8.17 (95% CI: 7.22–8.79).

## 4 Discussion

We examined the *in vitro* neurotoxicity of *N. naja* venom, and isolated and characterized short-chain and long-chain postsynaptic neurotoxins, i.e., α-Elapitoxin-Nn2a and α-Elapitoxin-Nn3a, respectively. Indian Polyvalent antivenom was able to prevent, but not reverse, the *in vitro* neurotoxicity of the short-chain α-Elapitoxin-Nn2a. In contrast, the *in vitro* neurotoxicity of the long-chain α-Elapitoxin-Nn3a was partially reversed by either Indian polyvalent antivenom or repeated washing of the tissue. α-Elapitoxin-Nn2a demonstrated non-competitive antagonism at skeletal nAChR when tested against concentration-response curves to CCh, while α-Elapitoxin-Nn3a displayed reversible competitive antagonism.

*N. naja* venom showed postsynaptic neurotoxicity in the indirectly stimulated chick biventer nerve-muscle preparation as indicated by the concentration-dependent inhibition of indirect twitches and reduction in contractile responses to external nicotinic agonists. This observation is consistent with proteomic analyses indicating a large abundance of postsynaptic neurotoxins and absence of pre-synaptic neurotoxins in the venom (Wong et al., 2021). The potency of the neurotoxic effects of the venom was moderate (i.e., t_90_ of 42 min at 10 μg/ml) compared with the potency of other elapid venoms we have previously studied using the same techniques. e.g., Australian inland taipan (*Oxyuranus microlepidotus*), 21 min at 10 μg/ml (Barber et al., 2014); Australian coastal taipan (*O. scutellatus*), 81 min at 10 μg/ml (Barber et al., 2014), and considerably weaker than the potency recorded for Chinese cobra (*N. atra*) venom which had a t_90_ of 17 min at the same concentration (Liang et al., 2020).

α-Elapitoxin-Nn2a and α-Elapitoxin-Nn3a were isolated from *N. naja* venom using reverse-phase HPLC. They appear to be minor components of the venom in relative abundance, constituting approximately 1 and 3% of *N. naja* venom, respectively. Combined with the relative abundance of peak 1, the components of *N. naja* venom with postsynaptic neurotoxicity, constitute approximately 9% of the venom protein content. This closely matches the findings by Wong et al. (2021), reporting that short- and long-chain neurotoxins constitute approximately 10% of *N. naja* venom from Sri Lanka. The α-neurotoxins in peak 1 could not be further purified using our chromatographic methods.

α-Elapitoxin-Nn2a has a molecular mass of 7,020.0 Da and consists of 62 amino acids, containing 8 cysteine residues that make four disulfide bonds, which are traits consistent with those of short-chain neurotoxins (Chang et al., 1997; Qiumin et al., 2002; Wei et al., 2003; Yang, 1999). In contrast, α-elapitoxin-Nn3a has a molecular mass of 7,807.5 Da and consists of 71 amino acids, containing 10 cysteine residues making five disulfide bonds, which are traits consistent with those of long-chain neurotoxins. The protein sequence of α-Elapitoxin-Nn3a had only 3 amino acid differences when compared to α-cobratoxin (α-elapitoxin-Nk2a) from the venom of the Monocled cobra (*Naja kaouthia*) (Betzel et al., 1991). The high degree of homology in primary structure with other short- and long-chain toxins which have been shown to bind to nAChR (Silva et al., 2018) further supports this as the primary site of action of α-Elapitoxin-Nn2a and α-Elapitoxin-Nn3a.

As such, α-Elapitoxin-Nn2a and α-Elapitoxin-Nn3a both displayed postsynaptic neurotoxicity *in vitro* as shown by the concentration-dependent inhibition of indirect twitches and reduction in contractile responses to exogenous nicotinic agonists. The pA_2_ values of α-Elapitoxin-Nn2a and α-Elapitoxin-Nn3a were 8.01 and 8.17, respectively, indicating high potency of the toxins towards the avian nAChR. These data are supported by the t_90_ values for the toxins which were 16 ± 1 min and 23 ± 4 min for α-Elapitoxin-Nn2a (300 nM) and α-Elapitoxin-Nn3a (300 nM), respectively (i.e., significantly faster than the whole venom). In comparison, the t_90_ for α-Elapitoxin-Na1a, a short-chain neurotoxin from Chinese cobra venom, was 17 ± 2 min (Liang et al., 2020), which is almost identical to the short-chain neurotoxin α-Elapitoxin-Nn3a, at the same concentration. Potent α-neurotoxins from other elapid venoms also share a similar pA_2_, such as α-Elapitoxin-Na1a (8.21) and α-Elapitoxin-Ot1a (8.02), isolated from the Chinese cobra (*Naja atra*) venom and Western Desert Taipan (*Oxyuranus temporalis*) venom, respectively (Barber et al., 2016; Liang et al., 2020). The binding of α-Elapitoxin-Nn2a with nAChR was non-competitive as evident from the concentration-dependent non-parallel shifts, with a marked depression of maximum responses, of the cumulative concentration-response curves to CCh. Similar properties have been shown for some elapid short-chain α-neurotoxins (Barber et al., 2016), where we have used the term “pseudo-irreversible” to describe this interaction with the nAChR. This is based on the premise that the toxins are not covalently bound with the nAChR but dissociate slowly from the receptor therefore giving the appearance of being non-competitive. It is also possible that α-Elapitoxin-Nn2a may inhibit “downstream” signaling linked to the nAChR (e.g., voltage gated Ca^2+^ channels) which contribute to the inhibition observed although this requires further investigation. Indian polyvalent antivenom was able to prevent the neurotoxicity caused by α-Elapitoxin-Nn2a, indicating the presence of antibodies against the toxin. However, the antivenom was unable to reverse the *in vitro* neurotoxicity of α-Elapitoxin-Nn2a, confirming that α-Elapitoxin-Nn2a is a poorly reversible short-chain α-neurotoxin.

In contrast, α-Elapitoxin-Nn3a caused a parallel rightward shift of the cumulative concentration-response curves to CCh, indicating that the antagonism of the toxin is competitive reversible which is uncharacteristic for long-chain α-neurotoxins (Blacklow et al., 2011; Chang & Lee, 1963; Jones et al., 1999; Nirthanan et al., 2002; Sheumack et al., 1990). In addition, the Indian polyvalent antivenom was able to prevent, as well as partially reverse, the neurotoxicity caused by α-Elapitoxin-Nn3a. Continuous physical removal of the toxin from the preparation by repeated washing also partially reversed the neurotoxicity caused by α-Elapitoxin-Nn3a, further confirming the uncharacteristic reversible nature of this long-chain α-neurotoxin. The reversibility or irreversibility of α-neurotoxin-induced neuromuscular blockade cannot simply be explained by the binding affinities of the toxins to muscle nAChRs, and could be associated with an area distinct from its pharmacophore, interfering with the association and dissociation of the toxin from the nAChR (Nirthanan, 2020). It has been long postulated that the difference in the reversibility of long-chain and short-chain α-neurotoxins could be related to the former having more hydrophobic residues in the primary structure (Lee et al., 1972; Barber et al., 2016). However, the structural insights into the uncharacteristically high reversibility of α-Elapitoxin-Nn3a warrants further investigation and was not an objective of the present study.

Although α-Elapitoxin-Nn2a is shown to be poorly reversible/non-competitive at avian nAChRs, it has been suggested that short-chain α-neurotoxins are unlikely to cause human paralysis because they dissociate readily from human nAChRs (Silva et al., 2018). In contrast, the clinical importance of long-chain α-neurotoxins has been postulated to be due to their higher potency and lower reversibility at human nAChRs. It is therefore promising that antivenom reverses the neuromuscular blockade by α-Elapitoxin-Nn3a. This may explain the successful treatment of bites from Asiatic cobras, which have large quantities of long-chain α-neurotoxins in their venoms (Lagraulet, 1984; Veto et al., 2007). Interestingly, a recent study has shown that the venom of the Samar cobra (*N. samarensis*) consists of approximately 66% short-chain α-neurotoxins, no long-chain α-neurotoxins were identified, and envenoming by this species causes paralysis in humans (Palasuberniam et al., 2021). This is an extremely high quantity of short-chain α-neurotoxins, and supports the hypothesis that short-chain toxins only cause envenoming in humans if administered in large quantities (Silva et al., 2018).

## Data Availability

The datasets presented in this study can be found in online repositories. The names of the repository/repositories and accession number(s) can be found below: https://www.uniprot.org/uniprot/, C0HM08; C0HM09.
