# Supplementary material for: Isolation and Characterization of Two Postsynaptic Neurotoxins From Indian Cobra (Naja Naja) Venom
Source: Front Pharmacol. 2022 Mar 28;13:815079. doi: 10.3389/fphar.2022.815079 (PMC8996157; doi:10.3389/fphar.2022.815079)
Supplement: Supplementary file 1 [file Image1.pdf]

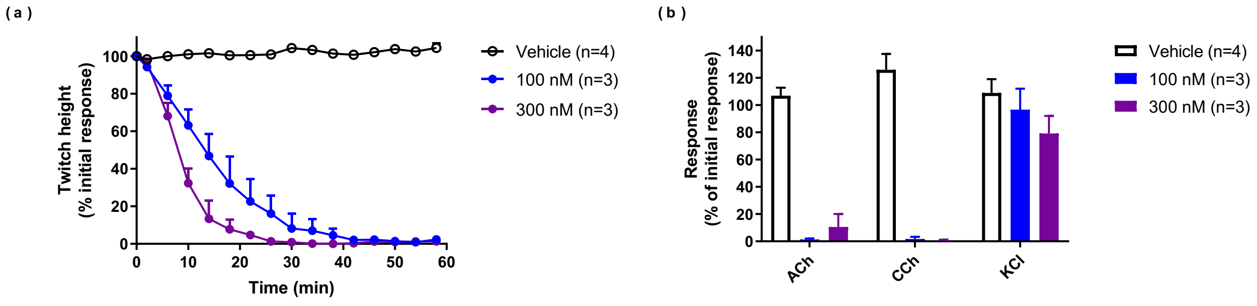

Supplementary figure 1. Concentration-dependent in vitro neurotoxicity of Fraction 1 (100-300 nM) on (a) indirect twitches and (b) responses to exogenous agonists ACh (1 mM), CCh (20 µM) and KCl (40 mM) in the chick biventer cervicis nerve-muscle preparation.

Although the fraction displayed post-synaptic neurotoxicity as evidenced by the inhibition of indirect twitches and responses to exogenous acetylcholine (ACh) and carbachol (CCh), further examination of this fraction was discontinued due to difficulties in obtaining a pure toxin.
